# Reliability of Root Canal Curvature Measurements and Canal Visibility Ratings over Repeated Calibration Rounds

**DOI:** 10.3390/dj14090553

**Published:** 2026-09-02

**Authors:** Mounir Ajjan Alhadid, Sabina Noreen Wuersching, Falk Schwendicke, Chantal Herbst, Sascha R. Herbst

**Affiliations:** Department of Conservative Dentistry, Periodontology and Digital Dentistry, University Hospital, LMU Munich, Goethestrasse 70, 80336 Munich, Germany

**Keywords:** angle, curvature, measurement, root canal, visibility

## Abstract

**Objectives:** To evaluate the inter- and intra-observer reliability of three methods for measuring root canal curvature and the reliability of canal visibility ratings on periapical radiographs over repeated calibration rounds. **Materials and Methods:** A total of 360 clinical periapical radiographs were divided into six batches and independently evaluated by three observers. Root canal curvature was measured using the Schneider, Weine, and a novel tangency-based method, while canal visibility was classified on a three-category scale. Inter- and intra-observer reliability were assessed using intraclass correlation coefficients (ICC). Agreement between observers was evaluated using Bland–Altman analysis, and differences between measurement methods were compared using repeated-measures analysis. The association between canal visibility and inter-observer measurement differences was assessed using Spearman rank correlation. **Results:** Inter-observer reliability for curvature measurements was predominantly good to excellent across all methods, with no consistent improvement over successive calibration rounds. Intra-observer reliability, assessed in one observer, was excellent (ICC 0.90–0.97). Measurement method significantly influenced angle values, with the Weine method producing larger angles than the Schneider and tangency-based methods, whereas the latter two showed comparable results. Agreement on canal visibility remained fair to moderate, and reduced canal visibility showed a weak but significant association with greater inter-observer differences in curvature measurements. **Conclusions:** All three methods demonstrated comparable inter-observer reliability, although the Weine method yielded systematically larger curvature angles. Agreement did not consistently improve over repeated calibration rounds. Reduced canal visibility may increase measurement variability and should be considered when interpreting root canal curvature on periapical radiographs.

## 1. Introduction

Root canal curvature and visibility are critical parameters that are regularly included in case-difficulty assessment tools embraced by international endodontic societies, such as the American Association of Endodontists (AAE) and the British Endodontic Society (BES) [1,2,3]. The rationale behind this is that curved root canals are especially prone to iatrogenic complications, including ledging, canal transportation, instrument separation, and perforation, during root canal treatment [4]. A recent study has also found that both canal curvature and visibility affect the technical outcome of root canal treatment, depending on the experience of the operator [5].

Despite its clinical importance, a standard method for measuring root-canal curvature based on radiographic images has not yet been achieved [6]. Several approaches have been proposed, with the most common being the techniques by Schneider [7] and Weine [8]. Some researchers argue that angle measurements are insufficient and propose to include other measures, like the radius of curvature [9,10] or canal access angle [11]. Lack of consensus prevents objective measurement and tends to make clinicians use personal preference, thus giving inconsistent and possibly subjective interpretations of canal trajectory. This inconsistency is compounded by variations in how professional societies classify curvature severity. For example, an angle of 10° might be considered as “mild” curvature by one system (BES) [2,3] but classified as “moderate difficulty” by another (AAE) [1]. The same differences can be observed in the assessment of the canal visibility, where the BES uses a five-stage scale [12], and the AAE uses a three-stage scheme.

A further challenge is that curvature assessment in routine care is commonly based on two-dimensional periapical radiographs. These images primarily represent the mesiodistal plane and may underestimate canal complexity by concealing curvatures in the buccolingual dimension [10,13,14]. While three-dimensional imaging (CBCT) can improve visualization of canal morphology, it is not recommended for routine use in every endodontic case; rather, periapical radiography remains the primary imaging method, and CBCT is generally reserved for situations in which 2D imaging is inadequate [15,16].

Another problem with measuring curvature is the aspect of observer interpretation, whereby various examiners may estimate the curvature angle differently. This is also true for estimating root canal visibility. In a study on the validity and reliability of an endodontic complexity assessment tool, Essam et al. [17] showed that these two parameters were the two most prevalent subjective variables that affected inter-rater reliability among general dentists. However, to date, the influence of canal visibility on the curvature angle measurement has not been investigated.

Calibration has been suggested as one way to reduce observer variability in radiographic interpretation, but existing evidence indicates that its effects may depend on the task. Past studies examining calibration effects on radiographic interpretation show mixed results: Sebring et al. [18] reported improved agreement for periapical lesion detection that partially decreased over time, while Meusburger et al. [19] found no significant increase in inter-rater agreement after calibration, despite improved intra-rater reliability. For measuring root canal curvature on routine radiographs, there is no objective standard; therefore, no measurement method can be considered a gold standard, and comparative work usually relies on agreement between methods or observers [20,21].

Therefore, the aim of the study was to provide a deeper understanding of the measurement of root-canal curvature and the assessment of canal visibility on clinical periapical radiographs. Three observers assessed several batches of clinical radiographs with three different measurement methods, namely Schneider [7], Weine [8], and a tangency-based method proposed by the authors. Canal visibility was rated on a three-stage scale. A batch-by-batch iterative calibration was used to examine patterns of inter-observer agreement across repeated calibration rounds and to identify systematic differences between the measurement methods. Intra-observer repeatability was additionally assessed for one observer in two batches.

## 2. Materials and Methods

This paper has been written in accordance with the GRRAS guidelines [22]. The study protocol was approved by Charité—Universitätsmedizin Berlin (Project No. EA4/080/18) and Ludwig-Maximilians-Universität München (Project No. 24/0337).

The required number of subjects was determined using the approximation proposed by Walter et al. [23] for intraclass correlation coefficient (ICC) reliability studies under a one-way random-effects model. The sample size calculation tested the hypothesis that reliability would meet an acceptable threshold. The null hypothesis assumed ICC = 0.60 (minimally acceptable), and the alternative hypothesis assumed ICC = 0.80 (expected level, based on Hartmann et al. [21]). With α = 0.05 (one-sided), power = 80%, and three observers evaluating the sample independently, the minimum required sample size was 27 images. In the present study, we included 360 periapical images.

A retrospective sample of 360 images was obtained from periapical radiographs exposed between 2016 and 2024 at the dental imaging department of the Charité Center of Oral Health Sciences. Images were acquired with Heliodent E dental X-ray units (Sirona, Bensheim, Germany) with a 305 mm (12-inch) tube and a 30.7 × 40.7 mm charge-coupled device (CCD sensor Sirona, Bensheim, Germany). The capture times were between 0.06 and 0.08 s, with 70 kV tube voltage and 7 mA current. Radiographs of permanent teeth with fully formed apices were chosen consecutively based on the following qualitative criteria: complete depiction of the tooth, absence of severe distortion, and sufficient contrast to delineate root contours. Teeth with developmental abnormalities, cervical caries, external or internal root resorption, previous endodontic treatment, or root fractures were excluded. The selected images were digitally separated to identify each tooth and then rotated vertically for standardized presentation and easier evaluation. The radiographs were then divided into six batches, each containing 34 molars, 20 premolars, and six anterior teeth.

Root canal curvature was measured using three methods: those proposed by Schneider [7] and Weine [8], and a novel method proposed by the authors. The techniques were adapted to S-shaped canals where necessary.

The Schneider method [7] involves drawing a line along the linear coronal portion of the root canal and another from the apex to the point where the canal deviates from the long axis. The angle is measured at their intersection. In the case of S-shaped canals, this method was modified to measure the angle between the cervical portion of the canal and a line between the end of the first curvature or the mid-third of the canal and the apex.

In Weine’s method [8], the coronal line was defined as a straight line fitted to the most coronal straight portion of the canal, aligned with the canal’s initial long axis. The apical line was defined as a straight line fitted to the most apical straight portion of the canal, extending from the apical foramen coronally until the canal deviated. In the case of S-shaped canals, this method was modified to include a middle segment defined by the curvature of the canal. Angles were then measured between this intermediate segment and the corresponding coronal and apical lines.

**Figure 1:**
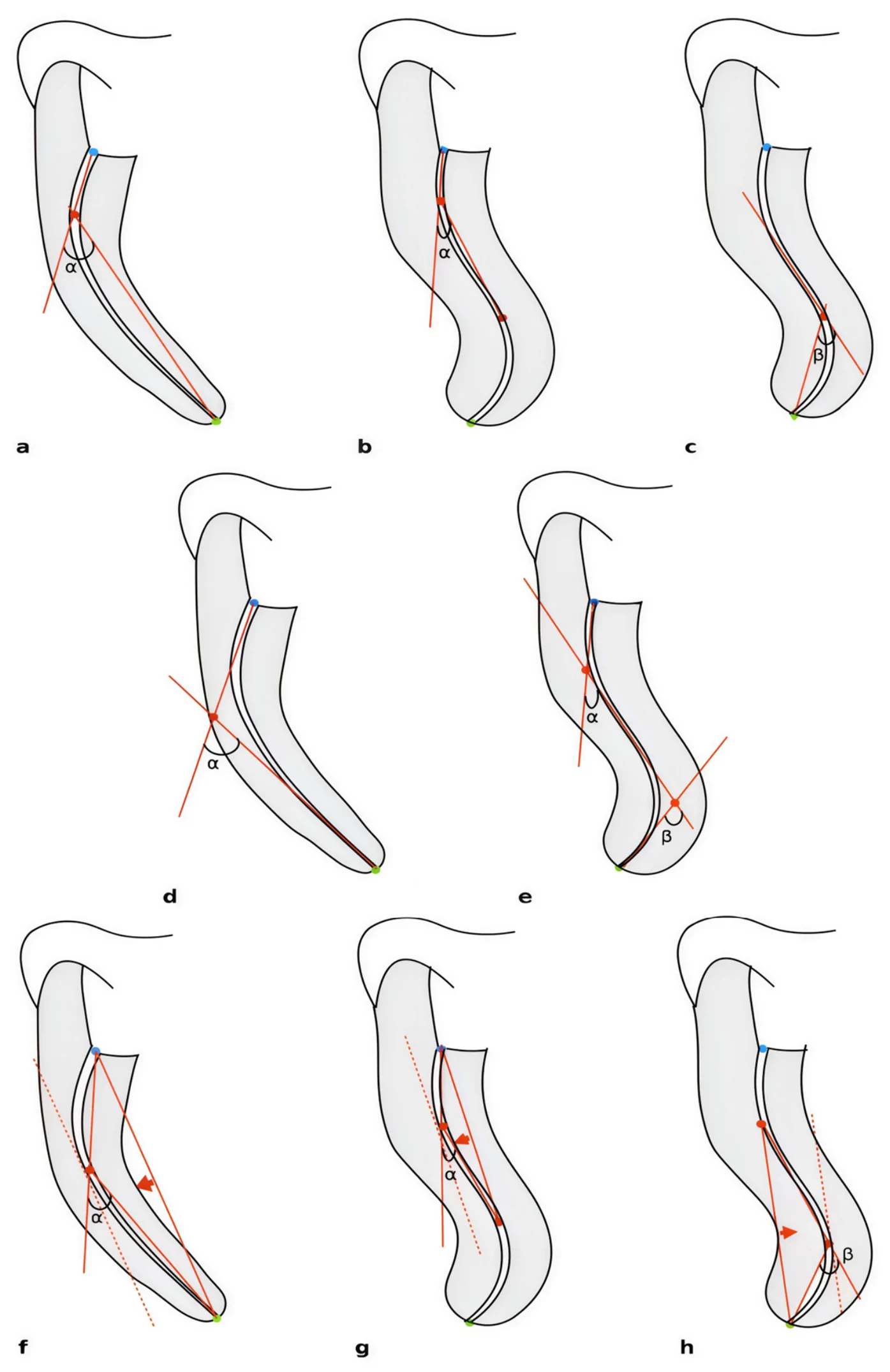
Schematic representation of curvature measurement methods: (**a**–**c**) Schneider, (**d**,**e**) Weine, and (**f**–**h**) the tangency-based method proposed by the authors, illustrated for single-curved and S-shaped root canals. The blue and green points indicate the canal orifice and apex, respectively. Red solid lines represent the method-specific reference lines, and red points mark the intersection or tangency points used to determine the curvature angles (α, β). In the tangency-based method (**f**–**h**), red dashed lines indicate the parallel-translated reference lines, and arrows showing the direction of translation.

A tangency-based method proposed in this study aims to provide a more geometrically defined procedure for determining reference points. It describes three main points of anatomical reference: the canal orifice, the apex, and the point of tangency of the canal curvature. A straight line is drawn from the canal orifice to the apex and translated in parallel until it is tangent to the canal. Two lines are drawn at this point of tangency, one towards the canal orifice and the other towards the apex. The curvature is defined by the angle between these two lines. In the case of S-shaped canals, two tangency points are determined; one of them is associated with the coronal curve and the other with the apical one. For these canals, lines are drawn between the canal orifice and the end of the first curve, the apex, and the beginning of the second curve. These lines are then translated in parallel until they are tangent to their respective curves. Angles are then measured at these tangent points. When the tangent line contacts the canal over a curved segment rather than at a single point, the midpoint of this segment is chosen as the tangency point. Conceptually similar geometric descriptors of canal curvature severity have been proposed previously by Kyomen et al. [24], although the present study defines curvature using angle measurements rather than curvature height. Schematic diagrams of each technique are given in detail in Figure 1.

Root canal visibility was rated as either visible, partially visible, or invisible. Visibility was rated as ‘visible’ for the entire canal length, ‘partially visible’ for only parts, and ‘invisible’ if the canal path was completely obscured in accordance with the Case Difficulty Assessment Form of the AAE [1].

Three dentists, MA (OB1), SW (OB2), and CH (OB3), with eleven, six, and three years of professional experience, respectively, served as observers for this study. OB1 holds a master’s degree in endodontics, while OB2 and OB3 are general dentists with a focused clinical interest in endodontics and routine experience in performing root canal treatment. A preliminary training phase with 25 images that were not included in the final data set was performed prior to formal data collection. This first exercise was designed to train observers in the application of the three methods of curvature measurement and the visibility classification system. The three observers rated the sample images independently. The observers were blinded to each other’s measurements until the finalization of the batches. The initial assessment step involved identifying the number of evaluable root canals and their geometry (e.g., S-shaped canals). The next step was to classify visibility and to measure the canal curvature using the RedBrick AI annotation tool (RedBrick AI, Inc., New York, NY, USA) [25]. In multirooted teeth, each root was evaluated separately. In maxillary molars, the mesiobuccal, distobuccal, and palatal roots were assessed independently; in mandibular molars, the mesial and distal roots were evaluated. When two canals were visible within the same root, the canal with greater curvature was selected independently by each observer for measurement. Discrepancies in canal selection were discussed during the subsequent calibration review. Visibility was not a reason to exclude a root canal; for partially visible or invisible canal segments, observers drew centrally placed best-fit lines within the expected canal trajectory in the annotation software, guided by the course of adjacent visible portions and the root outline. One observer (OB1) repeated all measurements in Batches 2 and 6 in two sessions, 3 weeks apart. The time interval between Batch 2 and Batch 6 was four months. This early–late repeat was added as an exploratory test of within-observer repeatability and possible drift (single observer in two batches).

RedBrick AI annotations, saved as a set of JSON files, were processed with Python 3.9 [26] to convert raw data into Excel files. R version 4.5.1 (R Foundation for Statistical Computing, Vienna, Austria) was used to conduct statistical tests.

Method differences were analyzed with repeated-measures ANOVA. Model assumptions were assessed by examining the normality of the residuals with the Shapiro–Wilk test (α = 0.05) and confirmed visually with Q-Q plots. Mean differences between the methods were obtained from estimated marginal means with Tukey adjustment (emmeans) with adjusted 95% confidence intervals (CI) and *p*-values.

Inter-observer reliability was calculated with the intraclass correlation coefficient (ICC) (2,1) (two-way random-effects, absolute agreement, single-measure), and intra-observer reliability was calculated with ICC (3,1) (two-way mixed-effects, absolute agreement, single-measure), both with 95% confidence intervals (CI). Interpretation followed Koo and Li: <0.50 poor, 0.50–0.75 moderate, 0.75–0.90 good, and >0.90 excellent [27].

Agreement on the classification of canal visibility was assessed using kappa statistics. The inter-observer agreement on classification among the three observers was estimated using Fleiss’ κ, and the intra-observer agreement (one observer, multiple observations in Batches 2 and 6) was estimated using Cohen’s κ. The kappa scores were interpreted using the Landis and Koch classification: <0.00 poor; 0.00–0.20 slight; 0.21–0.40 fair; 0.41–0.60 moderate; 0.61–0.80 substantial; and 0.81–1.00 very good [28].

Bland–Altman plots were used to illustrate inter-observer agreement by comparing the means of two observers pairwise, using the mean difference (bias) and 95% limits of agreement to identify systematic differences or outliers [29].

To explore whether image visibility influenced inter-observer variability in curvature measurement, the mean absolute differences between observers’ angle measurements per canal were calculated and correlated with canal visibility scores using Spearman’s rank correlation coefficient (ρ). Visibility was coded as invisible (0), partially visible (1), and visible (2). This analysis was exploratory.

After measuring each batch and conducting statistical analyses, the researchers jointly reviewed the cases identified as outliers in the Bland–Altman plots. These calibration reviews showed that the largest deviations in curvature angles arose from differences in interpreting the canal course and selecting landmarks under limited visibility, especially in anatomically complex situations such as S-shaped canals, apical deltas, or two traceable root canals within the same root sharing a track. Based on the problems identified during each calibration review, troubleshooting rules were agreed upon and documented to guide all observers in subsequent batches. This included measuring curvature only along the main canal path, ignoring apical deltas, recording a maximum of two curvatures per canal, and selecting the more curved canal in roots with two canals of similar visibility. The agreed rules were applied consistently by all observers and prospectively in subsequent batches; measurements already included in the statistical analyses for prior batches were not modified.

## 3. Results

A total of 360 cropped periapical radiographs were assessed by three observers in six batches. The total number of root canals defined as evaluable by consensus of the observers varied across the batches and ranged from 72 in Batch 2 to 92 in Batches 3 and 6. The number of S-shaped root canals varied from 13 in Batches 1 and 2 to 21 in Batch 6 (see Table 1). The angle measurement reliability between observers was predominantly good in all batches and methods, according to the criteria set by Koo and Li [27] (see Table 1). ICC values showed minor fluctuations between rounds but no consistent trend indicating improved agreement over time. By the final batch, Schneider, Weine, and the tangent method reached comparable inter-observer ICCs (0.82–0.85). Intra-observer reliability for one observer was excellent in both repeated batches (ICC: 0.90–0.97).

Agreement on canal visibility was lower than that of angle measurement. Inter-observer agreement across the batches ranged from fair to moderate. The highest Fleiss’ κ value was observed in Batch 2 (0.55, moderate agreement), while the lowest Fleiss’ κ was in Batch 3 (0.32, fair agreement), with angle measurement agreement also fluctuating in this batch. In the Batches 4 to 6, the values stabilized at a range of 0.38 to 0.41. Intra-observer agreement for visibility was substantial in Batch 2 (κ = 0.79) and only moderate in Batch 6 (κ = 0.45).

The comparison of curvature angles was performed using repeated-measures ANOVA, with F-values and the partial effect size (Cohen’s f) (see Table 2). The method factor was statistically significant in all analyses (*p* < 0.05), and Cohen’s f ranged from 0.45 to 0.96 in all batches and observers. The *F* values ranged from 16.7 to 94.6, showing a strong and consistent method effect on measured curvature.

Table 3 presents comparisons of the pairwise methods, which are corrected with the help of the Tukey post hoc procedure. The Weine method always produced significantly larger curvature angles than the Schneider method and the tangent method (adjusted *p* < 0.05 in all comparisons). The differences between the tangent and Schneider approaches were mostly minor and not significant, with one exception in Batch 2.

**Figure 2:**
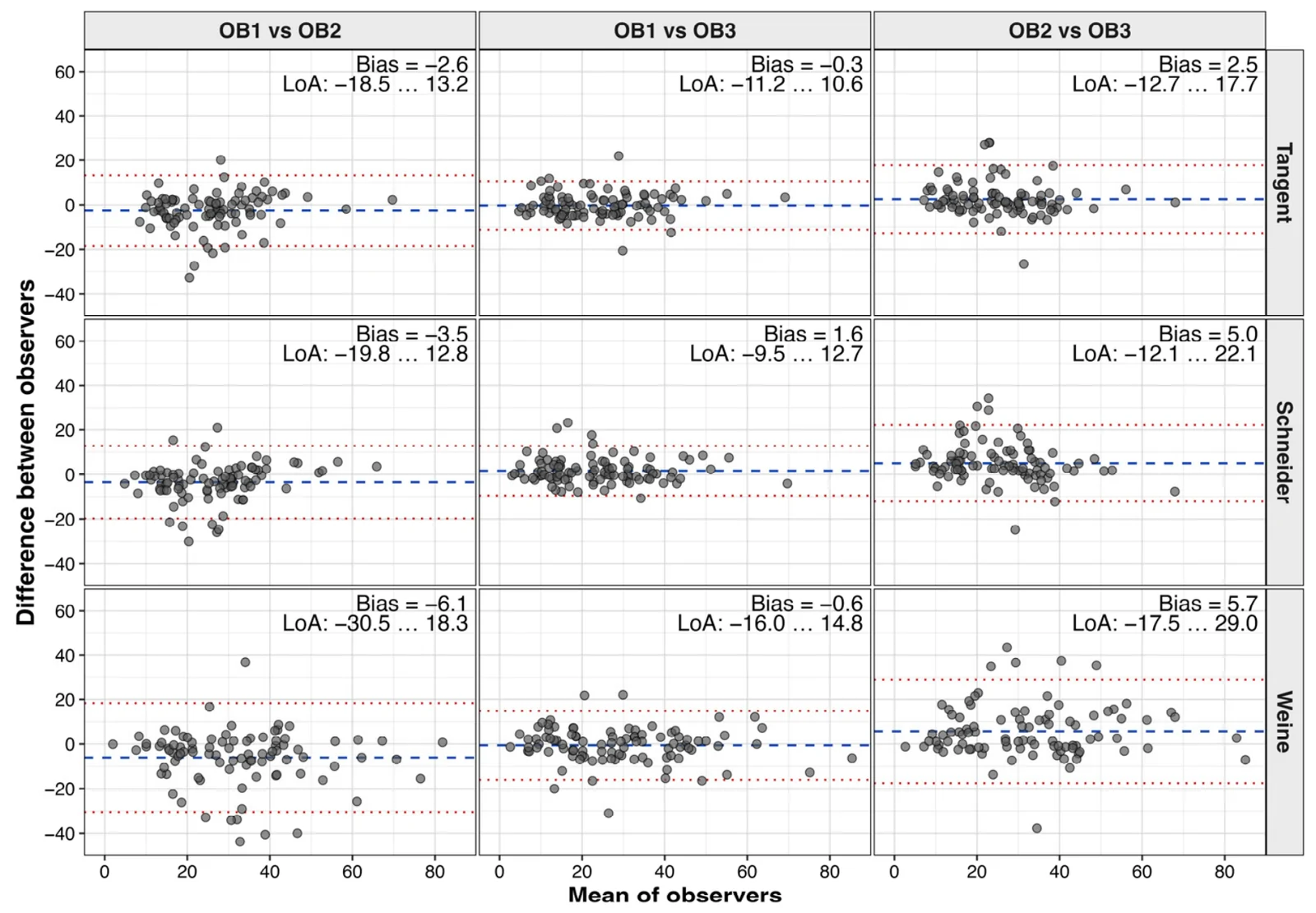
Bland–Altman plots showing pairwise inter-observer agreement for curvature measurements in Batch 1. The blue dashed line represents the mean difference, the red dotted lines indicate the 95% limits of agreement, and the black points represent individual observer-pair measurements. Complete panels are provided in the Supplementary Figures.

**Figure 3:**
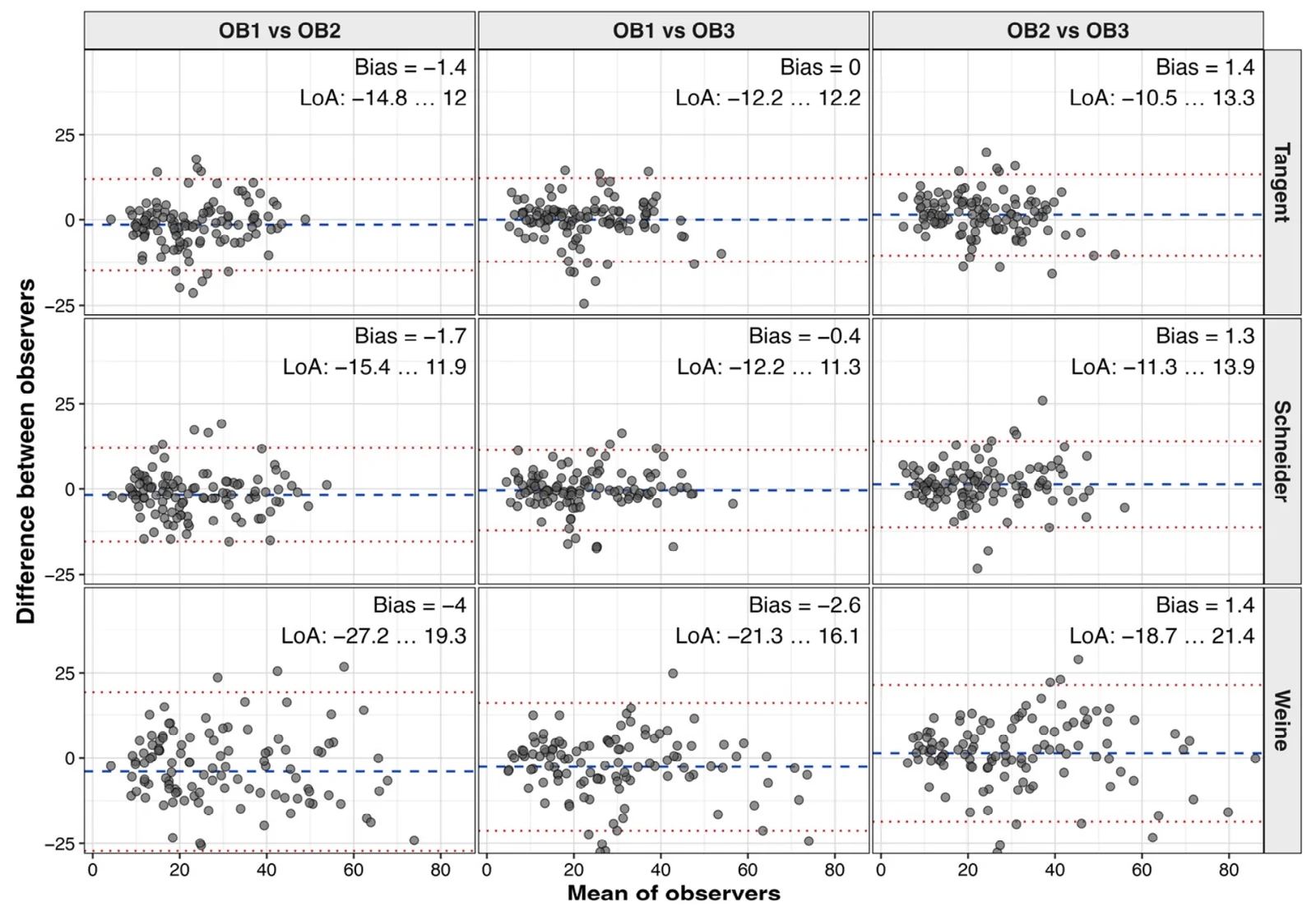
Bland–Altman plots showing pairwise inter-observer agreement for curvature measurements in Batch 6. The blue dashed line represents the mean difference, the red dotted lines indicate the 95% limits of agreement, and the black points represent individual observer-pair measurements. Complete panels are provided in the Supplementary Figures.

In addition to the ICC, pairwise observer agreement for curvature measurements was evaluated using Bland–Altman plots. Figure 2 and Figure 3 show the plots for the initial and final batches, respectively. Plots for Batches 2 to 5 and intra-observer comparisons are available in the Supplementary Materials. Across all batches, observed biases were generally small to moderate, ranging from 0° to 6° (see Supplementary Table S1). In Batch 1, the limits of agreement were relatively broad, especially for the Weine method, with stronger biases for the OB1 versus OB2 and OB2 versus OB3 observer pairs. In the following batches (see Supplementary Figures), the overall trend remained consistent, with narrower limits of agreement and reduced biases. By Batch 6, the limits of agreement for the Schneider and tangent methods had narrowed to approximately 11° to 12°, while the Weine method continued to show broader limits, around 20° to 30°. These results indicate slightly narrower limits of agreement in later batches, primarily for the Schneider and tangent methods, and confirm that the Weine method shows slightly greater dispersion among observers. Intra-observer Bland–Altman plots (see Supplementary Figures) were consistent with the excellent intra-observer ICCs, showing biases close to zero and narrow limits of agreement in both repeated batches. This indicates high within-observer stability.

Spearman correlation analysis revealed weak negative associations between canal visibility and inter-observer angle differences across all measurement methods (ρ = −0.12 to −0.14; *p* < 0.001; Table 4), indicating that lower visibility was associated with greater disagreement among observers.

## 4. Discussion

Although numerous methods exist for measuring root canal curvature, there is no consensus on the best approach [6]. This study is unique in that it compares the two well-established methods, Schneider’s [7] and Weine’s [8], and a newly proposed method using clinical periapical radiographs rather than radiographs of extracted teeth acquired in laboratory settings, and additionally evaluates inter-observer agreement in assessing root canal visibility. A key feature of our study is its iterative, multi-batch design, which allowed inter-observer agreement to be evaluated across repeated calibration rounds. However, our results did not demonstrate a consistent improvement in inter-observer agreement over time.

The inter-observer reliability of all three angle measurement methods was generally good and reached a comparable level by the final batch (ICC: 0.82–0.85). Although ICC values varied across batches, no consistent increase was observed. Because different radiographs were assessed in each batch, these fluctuations may reflect differences in case mix and anatomical complexity in addition to any effects of repeated calibration. Our results are consistent with Hartmann et al. [21], who reported ICCs of 0.815 for Schneider and 0.845 for Weine. Similarly, Faraj & Boutsioukis [20] found substantial to almost perfect inter-observer agreement (ICC: 0.76–0.82) when these two methods were used to measure angles. Hartmann et al. [21] examined the mesial roots of 41 mandibular first molars ex vivo. In contrast, we analyzed 360 images taken from clinical radiographs of varying quality, including S-shaped root canals across all tooth groups, using an iterative study design with repeated calibrations. Despite these basic differences in study design, we achieved similar reliability levels. This means that these measurement methods show stable reproducibility under clinical imaging conditions.

The Weine method consistently yielded higher angle measurements than the Schneider’s and the tangent methods. These findings are consistent with Zhu et al. [30], who reported Weine measurements that were approximately 8° larger than Schneider measurements, and with Mathew et al. [31], who reported systematically higher measurements for Weine compared to Schneider on both sagittal and coronal planes on CBCT images in permanent first mandibular molars. These differences were expected because each method has its own reference points and lines, and therefore, by definition, different angles are being measured. Curvature angle values obtained from different methods should not be directly compared, as doing so could result in misclassifying the severity of canal curvature.

The tangency-based approach was developed as a geometrically formalized modification of curvature assessment intended to reduce observer-dependent landmark selection. Whereas the canal orifice and apex provide anatomical reference points, the tangency point is geometrically derived. The mechanical relevance of this approach is supported by Pruett et al. [9] and Plotino et al. [32]: by design, the tangency-based angle aims at the segment of maximum canal curvature, where canal forces change direction, and rotating instruments are subject to peak bending stress and most prone to cyclic fatigue failure. However, the measurements obtained with this method were comparable to those obtained with Schneider’s method (mean difference: 0.13–2.45°), and it did not show an increase in inter-observer agreement. Thus, the additional complexity may limit its advantage for routine manual use. Nevertheless, the more formal geometric definition may be of interest for future computer-assisted or automated curvature assessment.

The Bland–Altman analysis confirmed the ICC reliability findings by showing small mean biases between observers without consistent systematic over- or underestimation. The limits of agreement were generally narrower for Schneider and the tangent method, but wider for Weine, indicating greater inter-observer variability when applying this method. When combined with the intraclass correlation coefficient (ICC) results, it appears that the choice of method has a greater influence on curvature measurements than the performance of the individual observers. Nevertheless, observer variability is still notable in borderline cases. Unlike Hartmann et al. [21], our study focused on observer-versus-observer reproducibility analysis for a particular method. In contrast, Hartmann et al. compared different methods using Bland–Altman analysis, which focuses on method-defined bias, so a direct comparison is not possible. However, we believe that the two approaches complement each other: the choice of method leads to systematic differences, whereas differences between observers using the same method are smaller and closer to zero.

Intra-observer reliability was assessed by a single observer (OB1) as a secondary objective of this study to evaluate consistency within the same observer over time. The ICC values were greater than those for inter-observer reliability (ICC: 0.90–0.97). These findings should therefore be interpreted as exploratory. Our results were consistent with Hartmann et al. [21], who found ICC values ranging from 0.90 to 0.94 for the more experienced evaluator.

Root canal visibility was assessed using a three-level categorical scale: visible, partially visible, and invisible, based on the Case Difficulty Assessment Form of the AAE [1]. The inter-observer agreement on canal visibility was fair to moderate (κ = 0.32–0.55) across batches, suggesting that training did not remove the differences between observers. The intra-observer agreement dropped from substantial in Batch 2 (κ = 0.79) to moderate in Batch 6 (κ = 0.45), confirming inconsistent visibility assessments. This variability in visibility assessment may also influence curvature measurement, particularly when best-fit lines are required for partially visible or invisible canal segments. However, the weak correlations observed in the present study indicate that visibility explains only a limited component of inter-observer disagreement.

Although statistically significant, the correlations between canal visibility and inter-observer angle differences were weak (ρ ≈ −0.12 to −0.14). This indicates that reduced radiographic visibility was associated with slightly greater measurement disagreement; however, the small effect sizes suggest that visibility alone accounts for only a limited proportion of the observed variability. Inter-observer differences in curvature measurement are therefore likely influenced by additional interpretative and methodological factors beyond image clarity.

The iterative multi-batch design enabled observation of the development of inter-observer agreement over time. While the Bland–Altman analysis generally indicated a reduction in the limits of agreement and a reduction in bias in later batches, ICC values did not consistently increase across the batches, suggesting that repeated calibration did not clearly improve inter-observer reliability. As this trend was not formally tested statistically, the observation should be considered indicative rather than conclusive. However, differences between batches may also be related to variations in the cases included in each batch, with anatomical complexity and image quality likely contributing to these variations.

From a clinical perspective, periapical radiographs remain the primary imaging modality for preoperative assessment. In our study, the three methods showed comparable reproducibility, but method choice significantly affected absolute angle values, which translates into different classification thresholds. For instance, the AAE classifies angles as <10° (low), 10–30° (moderate), >30° (high), whereas the BES classifies angles as <15° (mild), 15–30° (moderate), 30–60° (severe), >60° (exceptional). Our results did not show a clear superiority regarding the reliability of any single angle-measurement method but confirmed the systematic differences between techniques. From a diagnostic standpoint, one could argue that a method producing higher angle values, such as the Weine method, might offer more “security” in capturing the severity of the curvature since two-dimensional radiographs systematically underestimate the three-dimensional curvature. Mathew et al. [31] demonstrated that Weine’s method generally results in higher difficulty gradings than Schneider’s when classifying the curvature severity using the AAE Case Difficulty Assessment form and the EndoApp system of the BES.

All three methods in our study have the limitation of measuring only the angle between the coronal and apical canal segments and neglecting the middle third. Also, these methods do not represent the abruptness of the root canal curvature. Some authors have proposed the measurement of two angles, one between the coronal and middle segments, and another between the middle and apical segments [33,34]. Pruett et al. [9] showed that both the angle and radius of curvature are important in understanding canal complexity and predicting instrument stress during preparation.

The present study is original because it assesses the reliability of different methods using a large sample of 360 clinically acquired images, showing the robustness of angle measurement techniques under clinical imaging conditions. The multi-batch iterative design and continuous calibration between observers enabled the assessment of reliability limits. The inclusion of S-shaped roots, which are frequently underrepresented in research, allows assessment of reproducibility under anatomically complex and clinically relevant conditions that may challenge landmark identification on 2D radiographs. Additionally, our study uniquely evaluated inter- and intra-observer agreement on root canal visibility, a key requirement for curvature measurement that has received little attention in the literature despite its potential impact on measurement reliability. At this point, some limitations of the study must be acknowledged. The use of 2D periapical radiographs to represent 3D anatomy is a common practice in the clinic, but it lacks buccolingual information and may conceal relevant curvature. Accordingly, the present study evaluates the reproducibility of measurements obtained from two-dimensional radiographs and does not determine the accuracy of any method relative to the true three-dimensional canal anatomy. Moreover, superimposition and the radiograph’s projection angle may influence the apparent canal trajectory and centricity, which in turn could affect the placement of the landmarks. Standardizing the sample objects through cropping and vertical alignment may have made the assessment easier than with routine clinical radiographs, but it may reduce generalizability to unprocessed radiographs. The observers were dentists with experience in endodontic treatment and underwent repeated calibration. The observed reproducibility may therefore not be directly generalizable to clinicians with less experience or without comparable training. When canals were partially visible or invisible, observers were required to draw best-fit lines, which introduces some subjectivity despite calibration. The moderate to fair inter-observer agreement on visibility classification and the decrease in intra-observer agreement highlight that visibility assessment continues to be a challenge that impacts the reliability of subsequent curvature measurements. The study was conducted at a single center, which may limit generalizability to other centers and devices. Additionally, intra-observer repeatability was assessed by a single observer in two batches, and therefore, these results cannot be generalized across observers.

Our findings showed that the reproducibility of the angle curvature measurement is influenced by inter-observer variability. Reduced canal visibility was associated with slightly greater differences between observers; however, this association was weak and does not sufficiently explain the observed variability. While canal visibility may contribute to measurement differences, it cannot be considered the primary source of disagreement in our study. Investigators should therefore consider this aspect as one of several factors affecting curvature assessment. Advanced image enhancement techniques, such as Contrast Limited Adaptive Histogram Equalization (CLAHE), which improves local contrast while controlling noise amplification, may be relevant here [35]. The tangent method may also be of interest for automation because of its more formal geometric definition. Future research should investigate more curvature measurement methods and include features like the radius, canal height, or canal access angle. These efforts should also connect with the growing field of machine learning in clinical decision-making and treatment planning.

## 5. Conclusions

The three root canal angle measurement methods tested by three observers on clinical radiographs in an iterative study design showed good inter-observer agreement. The intra-observer reliability for a single observer was excellent. The Weine method produced consistently larger angles, while Schneider and the tangent method yielded similar results. The agreement on root canal visibility was fair to moderate. Reduced canal visibility was associated with slightly greater inter-observer differences in curvature measurements, although this effect was weak. Inter-observer agreement remained stable but did not consistently improve over repeated calibration rounds.

## Figures and Tables

**Table 1:** Inter- and intra-observer reliability (ICC), mean curvature angles, and κ for visibility classification. Inter-observer visibility agreement was assessed with Fleiss’ κ; intra-observer agreement (single observer, Batches 2 and 6) with Cohen’s κ.

Batch	*n*	S	Method	ICC (95% CI)	Mean Angle (°) ± (SD)			Visibility κ (95% CI)
					OB1	OB2	OB3	
			Schneider	0.79 (0.68–0.86)	24.6 (12.6)	22.9 (13.1)	28.0 (11.5)	
1	74	13	Weine	0.78 (0.68–0.85)	29.5 (16.2)	30.1 (18.1)	35.8 (17.4)	0.36 (0.25–0.47)
			Tangent	0.81 (0.74–0.87)	24.6 (12.1)	27.2 (12.0)	24.6 (11.1)	
								
			Schneider	0.84 (0.78–0.89)	23.8 (12.2)	22.9 (12.3)	25.7 (13.4)	
2	72	13	Weine	0.85 (0.78–0.90)	26.7 (16.2)	29.9 (17.5)	31.6 (19.5)	0.55 (0.43–0.67)
			Tangent	0.82 (0.77–0.87)	23.7 (12.2)	23.7 (11.3)	23.0 (12.9)	
								
			Schneider	0.92 (0.89–0.95)	24.0 (12.6)			
2 (INTRA)			Weine	0.92 (0.87–0.95)	29.4 (17.8)	-	-	0.79 (0.64–0.93)
			Tangent	0.90 (0.85–0.93)	24.4 (12.0)			
								
			Schneider	0.76 (0.69–0.82)	24.9 (12.6)	23.5 (11.8)	21.8 (12.7)	
3	92	16	Weine	0.84 (0.78–0.89)	32.7 (18.4)	31.5 (18.2)	29.2 (18.7)	0.32 (0.22–0.42)
			Tangent	0.72 (0.64–0.79)	25.2 (12.2)	22.7 (11.8)	22.1 (12.8)	
								
			Schneider	0.84 (0.78–0.88)	23.5 (11.1)	21.9 (10.0)	23.7 (11.4)	
4	89	17	Weine	0.87 (0.82–0.91)	29.5 (15.2)	28.3 (16.0)	31.9 (16.4)	0.38 (0.27–0.48)
			Tangent	0.79 (0.73–0.85)	23.5 (10.6)	21.8 (9.68)	23.3 (11.1)	
								
			Schneider	0.77 (0.70–0.83)	22.5 (12.9)	20.7 (11.8)	22.2 (11.6)	
5	89	16	Weine	0.80 (0.74–0.85)	27.7 (17.1)	26.0 (16.5)	28.7 (17.2)	0.41 (0.31–0.52)
			Tangent	0.78 (0.72–0.84)	21.8 (11.8)	21.3 (11.4)	21.9 (11.4)	
								
			Schneider	0.85 (0.80–0.89)	22.4 (11.8)	22.9 (12.0)	24.1 (11.7)	
6	92	21	Weine	0.82 (0.75–0.86)	28.3 (16.9)	30.9 (17.9)	32.2 (18.7)	0.40 (0.3–0.5)
			Tangent	0.82 (0.76–0.86)	22.6 (10.8)	22.6 (10.0)	24.0 (11.2)	
								
			Schneider	0.95 (0.93–0.97)	22.8 (12.1)			
6 (INTRA)			Weine	0.97 (0.95–0.98)	29.0 (17.3)	-	-	0.45 (0.32–0.59)
			Tangent	0.93 (0.90–0.95)	22.2 (11.2)			

Abbreviations: *n*, total number of root canals; S, number of S-shaped root canals; ICC, intraclass correlation coefficient; CI, confidence interval; SD, standard deviation; OB, observer; κ, Cohen’s or Fleiss’ kappa as indicated.

**Table 2:** Repeated-measures ANOVA results by batch and observer.

Batch	Observer	*F*	*f*
	OB1	46.74	0.74
1	OB2	78.91	0.96
	OB3	54.95	0.80
			
	OB1	16.74	0.45
2	OB2	60.84	0.86
	OB3	53.09	0.80
			
	OB1	60.23	0.75
3	OB2	94.6	0.94
	OB3	88.46	0.91
			
	OB1	59.87	0.76
4	OB2	81.25	0.89
	OB3	75.02	0.85
			
	OB1	55.22	0.74
5	OB2	82.43	0.90
	OB3	60.27	0.77
			
	OB1	55.07	0.70
6	OB2	83.75	0.86
	OB3	79.13	0.84

All repeated-measures ANOVAs indicated a significant effect of method (*p* < 0.05). Abbreviations: OB, Observer; *F*, F-statistic from repeated-measures ANOVA; *f*, Cohen’s f (partial effect size).

**Table 3:** Method comparisons (emmeans, Tukey) by batch and observer: mean differences in degrees (Δ°) with adjusted 95% CI and *p*-values. Negative values mean the first-listed method yields a lower angle than the comparator.

Batch	Observer	Weine—Schneider		Weine—Tangent		Tangent—Schneider	
		Δ° with 95% CI	*p*-Value	Δ° with 95% CI	*p*-Value	Δ° with 95% CI	*p*-Value
	OB1	5.0 (3.60–6.40)	<0.001	5.24 (3.79–6.69)	<0.001	0.23 (−1.21–1.68)	0.92
1	OB2	7.9 (6.05–9.75)	<0.001	9.0 (7.15–10.85)	<0.001	−1.11 (−2.96–0.74)	0.33
	OB3	7.2 (5.48–8.92)	<0.001	5.75 (4.03–7.47)	<0.001	1.46 (−0.26–3.18)	0.11
							
	OB1	3.51 (1.8–5.22)	<0.001	3.72 (2.01–5.43)	<0.001	−0.21 (−1.92–1.5)	0.95
2	OB2	6.52 (4.53–8.51)	<0.001	8.97 (6.98–10.96)	<0.001	−2.45 (−4.44–−0.46)	0.01
	OB3	8.28 (6.23–10.33)	<0.001	7.06 (5.01–9.11)	<0.001	1.22 (−0.83–3.27)	0.34
							
	OB1	7.82 (5.90–9.74)	<0.001	7.62 (5.7–9.54)	<0.001	0.19 (−1.72–2.11)	0.97
3	OB2	8.10 (6.41–9.79)	<0.001	8.94 (7.25–10.63)	<0.001	−0.84 (−2.53–0.85)	0.47
	OB3	7.54 (6.02–9.06)	<0.001	7.23 (5.71–8.75)	<0.001	0.24 (−1.28–1.76)	0.92
							
	OB1	5.85 (4.33–7.37)	<0.001	6.29 (4.77–7.81)	<0.001	−0.44 (−1.96–1.08)	0.77
4	OB2	8.42 (6.57–10.27)	<0.001	8.90 (7.05–10.75)	<0.001	−0.48 (−2.33–1.37)	0.81
	OB3	6.32 (4.85–7.79)	<0.001	6.86 (5.39–8.33)	<0.001	−0.54 (−2.01–0.93)	0.66
							
	OB1	5.19 (3.74–6.64)	<0.001	5.90 (4.45–7.35)	<0.001	−0.71 (−2.16–0.74)	0.48
5	OB2	6.53 (5.12–7.94)	<0.001	6.77 (5.36–8.18)	<0.001	−0.23 (−1.64–1.18)	0.92
	OB3	6.45 (4.93–7.97)	<0.001	5.69 (4.17–7.21)	<0.001	0.76 (−0.76–2.28)	0.46
							
	OB1	5.85 (4.35–7.35)	<0.001	5.72 (4.22–7.22)	<0.001	0.13 (−1.37–1.63)	0.98
6	OB2	8.08 (6.36–9.8)	<0.001	8.25 (6.53–9.8)	<0.001	−0.18 (−1.9–1.54)	0.97
	OB3	8.02 (6.25–9.79)	<0.001	8.32 (6.55–10.09)	<0.001	−0.30 (−2.07–1.47)	0.91

Abbreviations: Δ°, mean difference in curvature angle (degrees); CI, confidence interval; OB, observer.

**Table 4:** Spearman correlation between canal visibility and inter-observer angle differences.

Method	Spearman ρ	95% CI	*p*-Value
Schneider	−0.14	−0.21 to −0.05	<0.001
Weine	−0.12	−0.20 to −0.04	<0.001
Tangent	−0.13	−0.21 to −0.05	<0.001

Abbreviations: ρ, Spearman’s rank correlation coefficient; CI, confidence interval.

## Data Availability

The original contributions presented in this study are included in the article and Supplementary Materials. Further inquiries can be directed to the corresponding author.

## Supplementary Materials

The following supporting information can be downloaded at: https://www.mdpi.com/article/10.3390/dj14090553/s1, Supplementary Figure S1: Bland–Altman plots showing pairwise inter-observer agreement for curvature measurements in Batch 2. Supplementary Figure S2: Bland–Altman plots showing pairwise inter-observer agreement for curvature measurements in Batch 3. Supplementary Figure S3: Bland–Altman plots showing pairwise inter-observer agreement for curvature measurements in Batch 4. Supplementary Figure S4: Bland–Altman plots showing pairwise inter-observer agreement for curvature measurements in Batch 5. Supplementary Figure S5: Bland–Altman plots showing intra-observer agreement in Batch 2. Supplementary Figure S6: Bland–Altman plots showing intra-observer agreement in Batch 6. Supplementary Table S1: Bland–Altman statistics for inter-observer differences in curvature angle by batch, observer pair, and measurement method.

## Abbreviations

The following abbreviations are used in this manuscript:

AAE	American Association of Endodontists
BES	British Endodontic Society
CBCT	Cone-Beam Computed Tomography
CCD	Charge-Coupled Device
GRRAS	Guidelines for Reporting Reliability and Agreement Studies
ICC	Intraclass Correlation Coefficient
LoA	Limits of Agreement
LLoA	Lower Limit of Agreement
ULoA	Upper Limit of Agreement
OB	Observer
ρ	Spearman’s rank correlation coefficient
κ	Kappa coefficient

## References

[1] American Association of Endodontists (2022). Case Difficulty Assessment form and Guidelines. https://www.aae.org/specialty/clinical-resources/treatment-planning/case-assessment-tools/.

[2] Shah P.K., Chong B.S. (2018). A web-based endodontic case difficulty assessment tool. Clin. Oral Investig..

[3] Shah P.K., Duncan H.F., Abdullah D., Tomson P.L., Murray G., Friend T.M., Thomas S., Butcher S., Chong B.S. (2020). Comparison of two case difficulty assessment methods on cohorts of undergraduate dental students—A multi-center study. Int. Endod. J..

[4] Chaniotis A., Ordinola-Zapata R. (2022). Present status and future directions: Management of curved and calcified root canals. Int. Endod. J..

[5] Herbst S.R., Herbst C.S., Schwendicke F. (2023). Preoperative risk assessment does not allow to predict root filling length using machine learning: A longitudinal study. J. Dent..

[6] Hartmann R.C., Fensterseifer M., Peters O.A., de Figueiredo J.A.P., Gomes M.S., Rossi-Fedele G. (2019). Methods for measurement of root canal curvature: A systematic and critical review. Int. Endod. J..

[7] Schneider S.W. (1971). A comparison of canal preparations in straight and curved root canals. Oral Surg. Oral Med. Oral Pathol..

[8] Weine F.S. (1982). Endodontic Therapy.

[9] Pruett J.P., Clement D.J., Carnes D.L. (1997). Cyclic fatigue testing of nickel-titanium endodontic instruments. J. Endod..

[10] Schäfer E., Diez C., Hoppe W., Tepel J. (2002). Roentgenographic investigation of frequency and degree of canal curvatures in human permanent teeth. J. Endod..

[11] Günday M., Sazak H., Garip Y. (2005). A comparative study of three different root canal curvature measurement techniques and measuring the canal access angle in curved canals. J. Endod..

[12] British Endodontic Society (2022). BES Case Assessment Tool. https://bes-endoapp.typeform.com/bes-endoapp.

[13] Sousa T.O., Haiter-Neto F., Nascimento E.H.L., Peroni L.V., Freitas D.Q., Hassan B. (2017). Diagnostic Accuracy of Periapical Radiography and Cone-beam Computed Tomography in Identifying Root Canal Configuration of Human Premolars. J. Endod..

[14] Wu L., Ha W.N., Decurcio D.A., Estrela C., Rossi-Fedele G. (2023). Comparison of Curvature Severity Between Sagittal and Coronal Planes of Mesiobuccal Canals in Permanent Maxillary First Molars Using Multiple Complexity-risk Criteria: A CBCT Cross-sectional Study. J. Endod..

[15] Fayad M.I., Nair M., Levin M.D., Benavides E., Rubinstein R.A., Barghan S., Hirschberg C.S., Ruprecht A. (2015). AAE and AAOMR Joint Position Statement Use of Cone Beam Computed Tomography in Endodontics 2015 Update. Oral Surg. Oral Med. Oral Pathol. Oral Radiol..

[16] Patel S., Brown J., Semper M., Abella F., Mannocci F. (2019). European Society of Endodontology position statement: Use of cone beam computed tomography in endodontics. Int. Endod. J..

[17] Essam O., Boyle E.L., Whitworth J.M., Jarad F.D. (2021). The Endodontic Complexity Assessment Tool (E-CAT): A digital form for assessing root canal treatment case difficulty. Int. Endod. J..

[18] Sebring D., Kvist T., Buhlin K., Jonasson P., EndoReCo, Lund H. (2021). Calibration improves observer reliability in detecting periapical pathology on panoramic radiographs. Acta Odontol. Scand..

[19] Meusburger T., Wülk A., Kessler A., Heck K., Kühnisch J., Hickel R., Dujic H., Kühnisch J. (2023). The Detection of Dental Pathologies on Periapical Radiographs—Results from a Reliability Study. J. Clin. Med..

[20] Faraj S., Boutsioukis C. (2017). Observer variation in the assessment of root canal curvature. Int. Endod. J..

[21] Hartmann R.C., Ferraz E.S., Weissheimer T., Poli de Figueiredo J.A., Rossi-Fedele G., Gomes M.S. (2024). Comparative analysis of methods for measuring root canal curvature based on periapical radiography: A laboratory study. Int. Endod. J..

[22] Kottner J., Audigé L., Brorson S., Donner A., Gajewski B.J., Hróbjartsson A., Roberts C., Shoukri M., Streiner D.L. (2011). Guidelines for Reporting Reliability and Agreement Studies (GRRAS) were proposed. J. Clin. Epidemiol..

[23] Walter S.D., Eliasziw M., Donner A. (1998). Sample size and optimal designs for reliability studies. Stat. Med..

[24] Kyomen S.M., Caputo A.A., White S.N. (1994). Critical analysis of the balanced force technique in endodontics. J. Endod..

[25] Zantula Inc. RedBrick AI. https://www.redbrickai.com/.

[26] van Rossum G., Drake F.L. (2009). Python 3 Reference Manual.

[27] Koo T.K., Li M.Y. (2016). A Guideline of Selecting and Reporting Intraclass Correlation Coefficients for Reliability Research. J. Chiropr. Med..

[28] Landis J.R., Koch G.G. (1977). The Measurement of Observer Agreement for Categorical Data. Biometrics.

[29] Bland J.M., Altman D.G. (1986). Statistical methods for assessing agreement between two methods of clinical measurement. Lancet.

[30] Zhu Y.-Q., Gu Y.-X., Du R., Li C. (2003). Reliability of two methods on measuring root canal curvature. Int. Chin. J. Dent..

[31] Mathew A.I., Decurcio D.A., Estrela C., Wu L., Ha W.N., Rossi-Fedele G. (2026). Comparison of curvature severity between sagittal and coronal planes of mesial canals in permanent mandibular first molars using Schneider’s and Weine’s methods and multiple complexity–risk criteria: A CBCT cross-sectional study. Int. Endod. J..

[32] Plotino G., Grande N.M., Cordaro M., Testarelli L., Gambarini G. (2009). Measurement of the trajectory of different NiTi rotary instruments in an artificial canal specifically designed for cyclic fatigue tests. Oral Surg. Oral Med. Oral Pathol. Oral Radiol. Endod..

[33] Willershausen B., Tekyatan H., Kasaj A. (2006). Roentgenographic In Vitro Investigation of Frequency and Location of Curvatures in Human Maxillary Premolars. J. Endod..

[34] Fuentes R., Farfán C., Astete N., Navarro P., Arias A. (2018). Distal root curvatures in mandibular molars: Analysis using digital panoramic X-rays. Folia Morphol..

[35] Obuchowicz B., Zarzecka J., Strzelecki M., Jakubowska M., Obuchowicz R., Piórkowski A., Zarzecka-Francica E., Lasek J. (2025). Improving Endodontic Radiograph Interpretation with TV-CLAHE for Enhanced Root Canal Detection. J. Clin. Med..

