# Supplementary material for: Reliability of Root Canal Curvature Measurements and Canal Visibility Ratings over Repeated Calibration Rounds
Source: Dent J (Basel). 2026 Sep 2;14(9):553. doi: 10.3390/dj14090553 (PMC13605332; doi:10.3390/dj14090553)
Supplement: Supplementary file 1 [file dentistry-14-00553-s001.zip › Supplementary Table S1 .pdf]

Batch		OB1 vs. OB2				OB1 vs. OB3				OB2 vs. OB3			
		Bias°	SD°	LLoA°	ULoA°	Bias°	SD°	LLoA°	ULoA°	Bias°	SD°	LLoA°	ULoA°
**1**	**Schneider**	-3.5	8.3	-19.8	12.8	1.6	5.7	-9.5	12.7	5.0	8.7	-12.1	22.1
	**Weine**	-6.1	12.4	-30.5	18.3	-0.6	7.9	-16.0	14.8	5.7	11.9	-17.5	29.0
	**Tangent**	-2.6	8.1	-18.5	13.2	-0.3	6.0	-11.2	10.6	2.5	7.8	-12.7	17.7
**2**	**Schneider**	-1.8	6.9	-15.3	11.8	1.0	7.1	-12.4	14.5	2.6	6.4	-9.9	15.0
	**Weine**	-4.8	8.6	-21.6	12.1	3.5	10.4	-23.8	16.9	0.8	8.8	-16.4	18.0
	**Tangent**	0.4	7.2	-13.7	14.6	0.0	7.1	-14.0	13.9	-1.4	6.3	-13.7	10.9
**3**	**Schneider**	1.4	9.1	-16.3	19.2	3.2	9.2	-14.8	21.2	1.7	6.8	-11.6	14.9
	**Weine**	1.2	10.8	-20.0	22.4	3.8	11.3	-18.4	26.0	2.3	8.8	-15.0	19.5
	**Tangent**	2.4	9.6	-16.4	21.1	3.0	10.2	-17.0	22.9	0.7	6.6	-12.2	13.5
**4**	**Schneider**	-0.3	6.7	-13.4	12.8	1.6	5.8	-9.8	12.9	1.7	5.8	-9.7	13.1
	**Weine**	-2.7	8.8	-19.9	14.5	1.0	8.3	-15.3	17.3	3.6	6.5	-9.2	16.4
	**Tangent**	-0.1	5.63	-11.2	10.9	1.1	6.2	-11.0	13.2	1.1	6.0	-10.7	12.9
**5**	**Schneider**	0.3	9.6	-18.5	19.1	1.8	6.9	-11.7	15.3	1.6	8.2	-14.4	17.7
	**Weine**	-0.9	11.9	-24.2	22.4	0.5	9.6	-18.3	19.3	1.9	10.9	-19.6	23.3
	**Tangent**	-0.2	8.6	-17.0	16.6	0.4	6.9	-13.0	13.9	0.7	7.7	-14.4	15.7
**6**	**Schneider**	-1.7	7.0	-15.4	11.9	-0.4	6.0	-12.2	11.3	1.3	6.4	-11.3	13.9
	**Weine**	-4.0	11.9	-27.2	19.3	-2.6	9.5	-21.3	16.1	1.4	10.2	-18.7	21.4
	**Tangent**	-1.4	6.8	-14.8	12.0	0.00	6.2	-12.2	12.2	1.4	6.1	-10.5	13.3

Supplementary Table S1. Bland–Altman statistics for inter-rater differences in curvature angle by batch, rater pair, and method. Positive bias indicates larger angles by the first-listed rater. Abbreviations: OB, observer. SD, standard deviation. LLoA, lower limit of agreement. ULoA, upper limit of agreement.
